# Commencements

**Published:** 1892-05

**Authors:** 


### COMMENCEMENTS.

### Philadelphia Dental College.

The twenty-ninth annual commencement exercises of the Philadelphia Dental College were held at the American Academy of Music, Philadelphia, Pa., on Thursday, February 25, 1892, at 8 P. M.

The number of matriculates for the session was two hundred and fifty-nine.

The address to the graduates was delivered by Professor S. H. Guilford, D.D.S., Ph. D., and the valedictory address by J. R. Coleman, D.D.S.

The degree of D.D.S. was conferred on the following graduates by Ex-Governor James A. Beaver, President of the Board of Trustees:

| NAME | RESIDENCE |
|---|---|
| Egerton S Allen | Nova Scotia |
| Courtland J Allen | Rhode Island |
| J Wilmot Angwin | Canada |
| H D Atkinson | Missouri |
| Vincent J Baggot | Rhode Island |
| Adolf Balcke | Germany |
| J D Ballard | New Jersey |
| Wm A Bartlett jr | Maine |
| Howard S Bath | Canada |
| Johannes A Baumgardt | Germany |
| Frank G Bedell | New York |
| Fred W Benz | New York |
| Neil H Bishop | Ohio |
| John A Blackett | Australia |
| Sylvester A Bourgeois | Louisiana |
| D L Bower | Pennsylvania |
| Arthur M Bowman | New York |
| Gertrude A Bright | England |
| Joseph Brooks | Canada |
| Edwin D Butterworth | New York |
| Frank J Bush | New York |
| Hugh F Calder | Nova Scotia |
| Charles Cameron | Canada |
| E E Cawood | Oregon |
| Arthur J Chilcott | Maine |
| John A Clarke | Canada |
| J Edwin Clark | Pennsylvania |
| J Robinson Coleman | Canada |
| Edward B Cottrell | New Jersey |
| Oscar B Crawford | Pennsylvania |
| Robert Crawford | Australia |
| Wilbur B Cresswell | Pennsylvania |
| J Maurice Crosby | Canada |
| Wm A Crow | Canada |
| Wm N Daniels | Massachusetts |
| Jacques S David | Roumania |
| Wm F Dohrmann | California |
| R E Duignan | New York |
| L F Eaton | Connecticut |
| Elma H Edgar | New York |
| David L Edwards | New York |
| Frank A Elson | Ohio |
| Edmund P Ennis | Canada |
| H H Erskine | Ohio |
| Deering J Fisher | Rhode Island |
| Christopher E Fletcher | Missouri |
| Charles A Frain | Canada |
| Carlos F Fuentes | Chili |
| Edwin Russell Gamble | Pennsylvania |
| Alexander J Gillis | Massachusetts |
| Chas T Gliden | Pennsylvania |
| Wesley Good | Missouri |
| Sidney W. Gordon | Canada |
| Leslie H Grant | New Jersey |
| F H Greusel A B | Michigan |
| John Grieder jr | New Jersey |
| Percy L Haight | New York |
| Herbert E Hall | British Columbia |
| Chas R Hambly | Illinois |
| R S Hanna | Canada |
| Richard C Hart | New York |
| Harrie Tralee Harvey | Michigan |
| W G Henry | Pennsylvania |
| W E P Hewitt | Canada |
| F B Hewett | New York |
| R Russell Hogue | Georgia |
| Thomas C Hutchinson | Iowa |
| John L Jamison | Ohio |
| Bertha M Jarrett | Pennsylvania |
| Wm Jones | New York |
| Elton E Jordan | Maine |
| Lewis H Kalloch | Rhode Island |
| H P Kenney | Canada |
| C J Kennerdell | Pennsylvania |

| NAME | RESIDENCE | NAME | RESIDENCE |
|---|---|---|---|
| Chas A Kendall | Canada | W Henry Povall | New York |
| James A Kent | Minnesota | J B Pressey | New Jersey |
| Frank W Ketner | Pennsylvania | J W Purdy | Canada |
| Howard Kingsbury | Pennsylvania | R L Randall | New York |
| E L Lane | Oregon | Adolph G Reinhardt | Pennsylvania |
| Edgar D Larkin | Pennsylvania | C H Reynolds | Pennsylvania |
| Albert W Lavelli | Connecticut | Henry W Richards | Utah |
| Arthur Lemieux | Canada | Duncan P Robertson | Canada |
| T Segall Levien | Russia | Jonas S Rosenthal | Pennsylvania |
| Samuel Lobenstein | Missouri | Louis L Ruppert | New Jersey |
| Charles N Lord | New York | David A Scobie | New York |
| Frank R Lord | New York | G W Schock jr | Pennsylvania |
| F M Lynch | Washington | Maurice P Searle | Ohio |
| James A Lynch | Massachusetts | Frederick F. Seavers | Minnesota |
| Peter McGill | New Jersey | W B Sherman | California |
| A P McInnis | Minnesota | J C Shields jr | Oregon |
| John J McKinstry | Pennsylvania | Ella R Shinn | New Jersey |
| A J L McKechnie | Canada | Edward Shotthafer | New York |
| James A McLaren | Canada | E R Simmons | Germany |
| P J Macdonald | Massachusetts | John L Spanogle | Pennsylvania |
| Hugh S Mackay | Canada | J Henry Stackhouse | Canada |
| Carl C Marggraff | Connecticut | Milo H Steele | Canada |
| W E Marshall | Canada | Robert J Stevens | Canada |
| Chas F Meacham | Vermont | Margaret E Taylor | Pennsylvania |
| L D Mitchell | Canada | Zane B Taylor | Pennsylvania |
| Harry C Moore | Delaware | D A Telfer | Wisconsin |
| E H Munger | Connecticut | Howard A Thomas | Pennsylvania |
| Walter B Ousley | Iowa | George K Thomson | Canada |
| E C Palmer | Nebraska | J Melville Thompson | New York |
| W Y Pearsoll | Pennsylvania | Frank L Warren | New York |
| Mme Marie Pedemonte | Austria | Hugh A Whytock | Utah |
| Oliver K Pellman | Pennsylvania | L N Wiley | Connecticut |
| Edward J Pierce | New York | Chas D Winsor | Rhode Island |
| Glenn F Pollard | New York | Wallace Wood jr | Louisiana |

Total, 142

### United States Dental College.

The second annual commencement exercises of the United States Dental College were held in Recital Hall, Auditorium, Chicago, Ills., March 24, 1892.

The opening address was delivered by W. B. Marcusson, A.M., M.D.; the doctorate address by J. J. M. Angear, A.M., M.D.; and the valedictory was delivered by G. C. Stephens.

The number of matriculates for the session was fifty-seven.

The degree of D.D.S. was conferred on the following graduates by the President:

| NAME. | NAME. |
|---|---|
| Preston E Galloway | George J Marie |
| Fred E Field | Herbert R Johnson |
| Edwin E Newlin | Franklin A Fry |
| Frank A Carr | C E Schuchert |
| Elmer O Sarber | James C Rupert |
| George C Stephens | Elmer G Smith |
| George H Richardson | Joseph K Evans |

Total, 14

### Baltimore College of Dental Surgery.

The fifty-second annual commencement exercises of the Baltimore College of Dental Surgery were held at the Lyceum Theatre, Baltimore, Md., Monday evening, March 21, 1892.

The annual oration was delivered by Rev. F. M. Ellis, and the valedictory by Philip Ernest Sasscer, of Maryland.

The number of matriculates for the session was one hundred and eighty-one.

The degree of D.D.S. was conferred on the following graduates by Prof. R. B. Winder, Dean of the College:

| NAME | RESIDENCE |
|---|---|
| Benj. Dorney Altemus | Pennsylvania |
| Charles Wesley Arird | Pennsylvania |
| John Neven Baker | Pennsylvania |
| Irwin Joseph Beach | Maryland |
| William James Beatty | Pennsylvania |
| Chas. Alberto Bland | North Carolina |
| Chas. Wallace Boucher | Maryland |
| Harvey V. Bradshaw | Pennsylvania |
| Baskerville Bridgforth | Virginia |
| Burt B. Brumbaugh | Pennsylvania |
| James C. Buchanan | Pennsylvania |
| Wm. Carpenter Callahan | New York |
| Walter Caldwell Carter | Missouri |
| Fred. Abraham Charles | Massachusetts |
| Charles Alvin Cochel | Maryland |
| Robert Steele Cole | North Carolina |
| Edwin Davis | Pennsylvania |
| Willey Clark Dawson | West Virginia |
| Jacob William Derlin | Maryland |
| John S. Donaldson, D.D.S. | Colorado |
| James R. Donaldson, D.D.S. | Colorado |
| Harry Donnan | Pennsylvania |
| Benjamin Franklin Dulaney | Texas |
| Nelson Henry Ehle | Minnesota |
| Mortimer Lewis Fay | New York |
| Howard Roswell Fonda | Vermont |
| Harley Brooks Ford | Canada |
| Fred. Allen Ford | New York |
| Alexander Francis | Maryland |
| Isaac Abner Frazer | California |
| John Newton Giddeons | Alabama |
| Richard Lee Gill | Maryland |
| Washington Irving Goodwin | Canada |
| Harry White Graham | Pennsylvania |
| Archer C. Griffith | California |
| Emile Grosheintz, D.D.S. | Switzerland |
| Clarkson Newberry Guyer | Colorado |
| George Felder Hair | South Carolina |
| Charles Elward Hamilton | Georgia |
| Wm. Irvine Hatch, B.A. | South Carolina |
| Julio Hidalgo | Venez'a |
| W. S. Holbrook | New Jersey |
| Frank Harper Jackman | Connecticut |
| Alexander Jekelfalusy | Wisconsin |
| George Marshall Jones | Iowa |
| Earnest Paul Keerans | North Carolina |
| Clinton Kenney | Connecticut |
| Frederick Henry Kestler | California |
| Edward Thomas Ketcham | California |
| Harry Wilson Knauff | Pennsylvania |
| Milo Dempsey Kottraba | Pennsylvania |
| Robert Milton Krebs | Pennsylvania |
| Joseph Edwin La Force | Oregon |
| Emmet T. H. Leonard | Mississippi |
| James Isaac Logan | Alabama |
| Wm. Samuel Long | North Carolina |
| Wm. Latimer Lowe | Pennsylvania |
| Henry Herbst Maloney, A.M. | Louisiana |
| Edgar Watts Marven | Canada |
| James Walker Moore | Canada |
| Simon Bernard Meyer | Maryland |
| Patrick McCabe | Australia |
| Chas. Covington McCloud | Louisiana |
| George Bradley McFarland | E. India |
| Wm. Henry McGraw | Pennsylvania |
| Peter Alexander McLean | New Jersey |
| Ellis MacDougall | New York |
| John Elisha Parker | Texas |
| Leo Arthur Pusey | Virginia |
| Edgar Knox Rainey | Georgia |
| Louis Ambrose Reinhart | Maryland |
| Isaac Lemuel Ritter | Pennsylvania |
| Robert Ivey Robertson | Canada |
| Ryland Otey Sadler | North Carolina |
| Philip Ernest Sasscer | Maryland |
| George Harvey Sayre | New York |
| Albert Scott Shackleford | Texas |
| Zadoc Prescott Shaw | Maine |
| John Hartwell Smith | Virginia |
| William Henry Stokes | New York |
| John Emerson Storey | Texas |
| James Thomas Stuart | Alabama |
| Fred. Wickham Sweezy | New York |
| Wm. Aydelotte Taylor | Maryland |
| Fred. Aubrey Taylor | Canada |
| Clarence Hervey Terry | Texas |
| William Poole Terry | Louisiana |
| Albert Galiton Tillman | Mississippi |
| Eduardo Vasquez | Guat'a |
| Henry Augustin Truxillo | Louisiana |
| William Henry Walters | Maryland |
| Thomas Frederick Warnes | New York |
| Joseph D. Whiteman | Pennsylvania |
| Edgar Lucis Wilder | Vermont |
| Benjamin Hicks Williams | Georgia |
| David Morris Wilson | New York |
| Charlie Hurvey Winburn | Georgia |
| James Isaiah Woolverton | New Jersey |
| Frederie William Wright | Canada |
| James Anderson Yates | Kentucky |
| Robert Irving Youngs | New York |
| Rudolph Louis Zelenka | Louisiana |

Total, 102.

### Southern Medical College—Dental Department.

The Fifth Annual Commencement Exercises of the Southern Dental College were held at Dan'l Give's Opera House, Atlanta, Ga., on February 29, 1892.

The number of matriculates for the session was ninety-eight.

The Valedictory was delivered by W. S. Trent, D.D.S., of Alabama.

The degree of D.D.S. was conferred by the President, T. S. Powell, M.D., on the following persons:

| NAME | RESIDENCE | NAME | RESIDENCE |
|---|---|---|---|
| W. E. Buy | Georgia | T. R. Jones | Georgia |
| C. M. Bess | North Carolina | E. N. Kibler | South Carolina |
| W. L. Bethea | Georgia | T. D. Leonard | South Carolina |
| W. A. Blazenzame | Georgia | G. R. Lovelace | South Carolina |
| C. C. Burbank | Texas | W. C. Morgan | Florida |
| H. W. Carpenter | Georgia | W. G. Mason | Georgia |
| W. L. Cason | Georgia | W. F. Moore | Texas |
| T. D. Coty | Louisiana | C. Mermilliod | Louisiana |
| W. B. Cone | Florida | J. C. Powell | Georgia |
| J. E. Cramer | Georgia | S. O. Poore | Georgia |
| J. H. Cates | Georgia | A. C. Parry | Georgia |
| J. L. Dean | South Carolina | A. A. Patterson | South Carolina |
| G. W. Davis | Georgia | R. A. Patterson | Georgia |
| W. A. Ellis | North Carolina | C. C. Parish | Georgia |
| O. E. Griffin | Georgia | T. B. Robbins | Alabama |
| Z. Greene | Alabama | J. R. Rountree | Georgia |
| G. K. Hawley | Connecticut | J. C. Smith | Georgia |
| W. L. Hightower | Alabama | D. M. Snelson | Georgia |
| J. G. Heard | Alabama | W. H. Spinks | Texas |
| J. J. Hendley | Georgia | W. A. Summerlin | Georgia |
| T. W. Henderson | Georgia | F. H. Smith | Mississippi |
| F. A. Henley | North Carolina | G. D. Stovall | Alabama |
| H. R. Jewett | Georgia | W. S. Trent | Alabama |
| A. M. Jackson | Georgia | W. E. Wheeler | Tennessee |
| R. P. Jackson | Georgia | J. R. Warren | Georgia |
| A. M. Jamerson | Georgia | Total, 51. | |

### Meharry Medical College—Dental Department.

The Sixth Annual Commencement of the Dental Department of the Meharry Medical College was held in connection with that of the Medical and Pharmaceutical Departments, at Nashville, Tenn., on February 18, 1892.

George W. Miller, of the Medical Class, gave the address of welcome, and J. W. Holmes delivered the Valedictory.

During the past session seven students have been enrolled in the Dental Department.

President J. Braden conferred the degree of Doctor of Dental Surgery on J. B. Singleton, of South Carolina.

### Missouri Dental College.

The twenty-sixth annual commencement exercises of the Dental Department of Washington University, were held in connection with those of the St. Louis Medical College, at Memorial Hall, St. Louis, Mo., on Thursday, March 10, 1892.

Prof. Wm. Townsend Porter delivered the address to the class.

The number of matriculates for the session was seventy-seven.

Chancellor Chaplin of the University conferred the degree of D.D.S. upon the following graduates:

| NAME | RESIDENCE | NAME | RESIDENCE |
|---|---|---|---|
| F. H. Achelpohl | Missouri | W. Kalbfleisch | Illinois |
| G. W. Applegate | Missouri | A. Lambert | Illinois |
| O. W. Bedell | Ohio | A. N. Milster | Missouri |
| J. L. Bridgford | Missouri | O. Mallinckrodt | Missouri |
| O. F. Burton | Missouri | A. T. Moser | Missouri |
| H. F. Cassell | Missouri | C. Muetze. | Missouri |
| I. B. Coil | Missouri | O. H. Manhard | Missouri |
| C. C. Cowdery | Ohio | H. F. Naumann | Missouri |
| W. G. Cox | Missouri | J. L. Perry | Illinois |
| W. G. Goodrich | Missouri | W. L. Pruett | Missouri |
| L. E. Gordon | Illinois | W. F. Schwaner | Iowa |
| C. G. Hampton | Missouri | E. Schaer | Switzerland |
| R. J. Hart | Wisconsin | T. Trotter | Missouri |
| T. D. Head | Missouri | W. G. Teel. | Virginia |
| E. M. Hurd | Nebraska | H. G. G. Van Aller | Germany |
| W. P. Inglish | Missouri | F. F. Worthen | Illinois |
| S. Jacoby | Missouri | Total, 33 | |

### Cincinnati College of Medicine and Surgery—Dental Department.

The first annual commencement of the Dental Department of the Cincinnati College of Medicine and Surgery, was held at the Y. M. C. A. Hall, on Wednesday evening, March 16, 1892.

The opening address was delivered by Prof. G. S. Junkerman, M.D., D.D.S., Dean of the Faculty; and the Valedictory by Prof. A. I. F. Buxbaum, M.D., D.D.S.

The number of matriculates for the session was thirty-four.

The degree of D.D.S., was conferred on the following graduates by Prof. Geo. W. Harper, President of the Board of Trustees:

| NAME. | RESIDENCE. | NAME. | RESIDENCE |
|---|---|---|---|
| Sam. H. Wardle | Ohio | Fred. G. Williams | Ohio |
| B. Frank Corwin | Ohio | Ernest Bragdon, M. D | Ohio |
| C. E. Sibbet | Ohio | Wm. Francis Sloan | Pennsylvania |
| J. F. Clayton | Ohio | G. W. Hoffman | Ohio |
| James Franklin McCamant | Ohio | John C. Wallace, M. D | Michigan |

Total, 10

### Chicago College of Dental Surgery.

The tenth annual commencement exercises of the Chicago College of Dental Surgery (Dental Department of the Lake Forest University) were held at the Columbia Theatre, Chicago, Ill., on Tuesday, March 22, 1892, at 2:30 P. M.

The annual address was delivered by W. C. Roberts, D.D., LL.D., President of the University, and the doctorate address by C. N. Johnson, L.D.S., D.D.S.

The number of matriculates for the session was three hundred and three.

The degree of D.D.S. was conferred on the following graduates by Truman W. Brophy, M.D., D.D.S., Dean of the College:

| NAME. | NAME. |
|---|---|
| Albert Bromley Allen | George Emil Franke |
| Clarence Edson Allshouse | Edward M S Fernandez |
| George Henry Anderson | George Ramsey Guild |
| Gustave Edward Anderson | John J Geary |
| Hubbard Gail Atwater | Robert Good |
| Ernest Allin | Jeremiah Gochenour |
| Manning Andrus Birge | Francis Marion Gray |
| Thomas Jefferson Borland | Alfred J Homfeld |
| Jabez Bunting Burns | Julian Frank Hixon |
| John L Bingham | Augustus Finley Henning |
| Lemuel Fairfax Buck | A Gallagher Hebberd |
| Calvin Fergeson Besore | Marion L Higgins |
| Albert Leslie Bents | Robert Anderson Howell |
| Samuel Hardesty Baker | Fred Armstrong Ironside |
| Mark Robert Brierly | Albert Hamilton Johnston |
| Benjamin Dornblazer Barber | Albert George Johnson |
| John William Beetham | Austin Flint James |
| Frank Carlton Colby | Frank King |
| Charles Robert Currier | Ernest Venzel Kautsky |
| Harlow Arthur Cross | William Frederick Leu |
| Curtis Hammond Coe | Lewis Schuyler LaPierre |
| Frank L Condit | Frank Leslie Lane |
| Bert C Campbell | William Arthur Lewis |
| John Corwin | Hallvard Lie |
| Robert Clark Coy | William Cutler Lumpkin |
| Herbert Armstrong Carson | Oscar Edward Meyer |
| Orie George Collins | John Franklin McCrea |
| Albert Paul Condon | William Ephraim Martin |
| Amos Winship Dana | Herman Minges |
| Will Conger Dunn | John Simpson McQueen |
| James House Davis | John Benedict Mason |
| Claude Howard Devereaux | Henry Bruce Meade |
| Frank Elmer David | Walter John Morrow |
| Hiram Darling | John Henry Muenster |
| Lewis Mathias Doerr | Samuel Alexander Nielson |
| Henry Wallis Ewing | Bert Newsome |
| Albert Eugene Eagles | James Toberman New |
| William Edgar Ervin | John Egbert Nyman |
| Walter Howard Fox | Charles H Oakman |
| Lewis Eugene Ford | Charles Fremont Palmer |
| Allen Joseph Freeman | Albert James Prescott |
| Frank Orin Finley | James Lyon Palmer |
| Herman Peter Fischer | William Abraham Penn |
| Lawrence Sylvester Fezer | Franklin Pfeiffer |

NAME

George Thomas Page
John Dominic Purcell
William Conover Parsons
Frank Everett Phillips
George Samuel Root
Dennis Herbert Rowells
Fred Emerson Reynolds
Charles Bennett Reynolds
James Arnold Reynolds
William Woods Robbins
Joseph Herbert Robinson
Victor Hugo Rea
Edgar Miner Richards
Robert Hutchison Robertson
Omro Elmer Severance
Bertram Grey Smith
Ard Patterson Smith
Fred John Staehle
George McKay Sutherland
Sylvester Elmer Stouffer

NAME

John Franklin Stephan
James Byron Stuck
Jeffrey Springle
Carl Oscar Wilhelm Schycker
Sebastian Ricardo Salazar
Paul Steinberg
Arthur George Tibbitts
Richard Elmer Thexton
Edgar Felker Thomas
Herbert Hawkesworth Tyler
Edward Robert Victor
Matthew Wilson
Hans Bastian Wiborg
James Abram Welsch
Clarence Walter Williams
Charles Albert Wedge
Fred H Wallace
Frank Pierce Welch
William F Whalen
Louvain Alden Werden

Total, 128

### Kansas City Dental College.

The Tenth Annual Commencement of the Kansas City Dental College was held in Grand Avenue M. E. Church, Kansas City, Mo., on March 4, 1892.

The Faculty address was delivered by Prof. Theo. Stanley, and an address was also delivered by Rev. J. E. Roberts.

The number of matriculates for the session was eighty-seven.

The degree of D.D.S. was conferred on the following graduates by Dr. L. C. Wasson, President of the College Association:

NAME

Charles William Day
David Kerr Bryson
Harry Baile Engel
Harry Mitchell Doyle
Charles Willetts Thompson
Henry Wilfred Kelly
Alanson Tuttle Havely
William Amos McKee
Frank M Blake
Johann Christian Buttner
Ernest Prindel Noble
Jefferson Davis Barton
Harry Hurt Turner
Robert Edgar Barton
Oliver Tennyson Griner
Arthur Hoffman Bagby
John Malcolm Campbell
Frank Lincoln Williams
James Daniel Neff
Irwin Wilson Dills
James William O'Bryon
James Whitehill Butt
John Howell Jenkins
Ned Elmore White
John George Alexander Kydd

NAME

Arthur Monroe Tutt
Martin Henry Hopfer
George Leon Tetrick
Samuel Joseph Renz
John Bratton Woodside
Walter Emmitt Highnote
Daniel Franklin Pendleton
George Washington Amerman
Ludwig Henning Bredouw
Fred Lewis Cobb
Frank Lenoir Carter
Mark Chester Lovell
James Henry Goodwin
Gustavus Montgomery Cross
Amasa Molton Farnham
Woodson Thompson Smith
Ole Anderson Smith
Schuyler Colfax Grant
Fred Pierce Cronkite
George Daniel Mitchell
Pitts Elmore Wilhite
Clifford Howell Nelson
Henry Eugene Lindas
Arthur Lee Lindsey
Eugene Aquilla Chase

Total, 50

### University of Iowa—Dental Department.

The Tenth Annual Commencement of the Dental Department of the State University of Iowa was held in the Armory, Iowa City, Iowa, on Thursday evening, March 10, 1892.

The annual address was delivered by John J. R. Patrick, M.D., D.D.S.

The degree of D.D.S. was conferred on the following persons by the President, Charles A. Schaeffer, Ph. D. :

| NAME | RESIDENCE |
|---|---|
| M F Anderson | Muscatine |
| Fred Anderegg | Mankato, Minn |
| H W Anger | Brooklyn |
| R N Baker | Iowa City |
| J W Ball | Delaware |
| F A Boysen | Dubuque |
| Hugo Braun | Davenport |
| H W Baldwin | Oconomowoc, Wis |
| M Brennan | Ashland, Wis |
| J C Braley | Harvey, Ills |
| C P Burt | Elwood, Ills |
| E H Ball | Philips, Neb |
| M H Breen | LeMars |
| J F Curry | Friendship, N Y |
| F P Chapman | Clarinda, Ia |
| W A Dredge | Amboy, Minn |
| C F Dwight | Marcus |
| E S Denbo | Corydon, Ind |
| L A Grigsby | Lena, Ills |
| J C Holson | Iowa City |
| J C Hullinger | DeWitt |
| W S Hosford | Iowa City |
| M A Humphrey | Minona, Ia |
| T B Heckert | Red Oak, Ia |
| D A Haines | Decorah |
| L G Holmes | Birmingham |
| H M Harlan | Seymour |
| M A H Jones | Iowa City |
| C H Jacobs | Colesburg |
| Harry Kelso | Ames |
| E W Kerr | Newton |
| C B Miller | Waterloo |
| J G McCartney | Mitchell, S Dak |
| H C McCrea | Greenfield |
| E S McWhorter | Canon City |
| Miss O A Otte | Peabody, Kas |
| Miss Julia Otte | Peaboby, Kas |
| Miss A E Owens | Parkersburg |
| W H Pallett | Dorchester, Neb |
| W W Perry | Elizabeth, Ills |
| B A Price | Afton |
| H R Pasedach | Tipton |
| G F Pratt | Red Oak |
| E A Rogers | Vinton |
| H F Randolph | Belle Plaine |
| G H Reynolds | Binghamton, N Y |
| H C Shoemaker | Muscatine |
| Wm Schlawig | Sioux City |
| W G Skidmore | Moline, Ills |
| J E Stinehart | Iowa City |
| T S Stanford | Cambridgeboro, Pa |
| S R Swain | Iowa City |
| O H Sossaman | Waterloo |
| F A Strayer | Jefferson |
| M W Warner | Parkersburg |
| R J Wilson | Oelwein |
| F R Wright | Morning Sun |

Total, 57

### Royal College of Dental Surgeons of Ontario.

The number of matriculates for the session at the Royal College of Dental Surgeons of Ontario was sixty-four.

After the examination the title of L.D.S. (Licentiate of Dental Surgery) was conferred upon the following :

| NAME | RESIDENCE |
|---|---|
| S A Aykroyd | Kingston |
| R Agnew | Clinton |
| S Anderson | Mitchell |
| E A Billings | Leamington |
| J A Black | Kingston |
| W A Burns | St Thomas |
| J A Edwards, D.D.S | Uxbridge |
| J H Fell | Burlington |
| Herman Hart | Lindsay |
| H F Kinsman | Exeter |
| F A Lackner, D.D.S | Berlin |
| H C Lake | Toronto |
| M A Morrison | Peterboro |
| G J Musgrove | Wingham |
| F D Price | Napanee |
| F B Ross | Hamilton |
| D C Smith | Uxbridge |
| T C Trigger | St. Thomas |
| G A Walters | Forest |
| W R Wilkinson, D.D.S | Elmira |
| S C Wilson, D.D.S | Illinois |

Total, 21

### Columbian University—Dental Department.

The Fifth Annual Commencement Exercises of the Dental Department of the Columbian University were held, in connection with those of the Medical Department, at Albaugh's Opera House, Washington, D. C., on Thursday, March 17, 1892, at 2:30 P. M.

The address to the Dental Graduates was delivered by John B. Rich, D.D.S.

The number of matriculates for the session was thirty-five.

The degree of D.D.S. was conferred on the following graduates by President J. C. Welling, LL. D.:

| NAME | RESIDENCE | NAME | RESIDENCE |
|---|---|---|---|
| John H Burch | District of Columbia | Geo H Townsend | Virginia |
| Wm L Clark | District of Columbia | Carl Trede, M.D | Germany |
| Alva Sigel Roush, A.M., M.D. | Ohio | Total, 5 | |

### Western Dental College of Kansas City.

The Second Annual Commencement of the Western Dental College was held in the Music Hall, Kansas City, Mo., on the evening of March 10, 1892.

The Faculty address was delivered by Prof. H. O. Hanawalt.

The number of matriculates for the session was seventy-nine.

The degree of D.D.S. was conferred on the following graduates:

| NAME | RESIDENCE | NAME | RESIDENCE |
|---|---|---|---|
| L P Austin | New York | Otto Jacobs | Missouri |
| K P Ashley | Kansas | O J Keuper | Missouri |
| W C Allen | Missouri | C B Leavel | Missouri |
| E C Brownlie | Missouri | I B Nordyke | Missouri |
| A C Barr | Illinois | P J Oreiley | Missouri |
| W H Condit | Kansas | S T Peter | Nebraska |
| T H Cunningham | Missouri | C Robertson, jr | Kansas |
| F W Drom | Nebraska | J F Spence | Missouri |
| R E Darby | Missouri | J H Swan | Missouri |
| Fred W Franklin | Missouri | H H Sullivan | Missouri |
| F E Gaines | Missouri | W W Simpson | Kansas |
| T J Herkins | Kansas | A L Smith | Missouri |
| T I Hattield | Kansas | E C Taylor | Missouri |
| D J Hayden | Kansas | M D Vanhorn | Illinois |
| Wm Harrison | Nebraska | O C West | Missouri |
| C C Jones | Kansas | Frank S Webster | Kansas |
| L G Jones | Kansas | A S Wright | Missouri |
| S E Johnson | Kansas | H Yant | Kansas |
| F W Johnson | Missouri | Mrs Alice Yant | Kansas |

Total, 38

### Tennessee Medical College—Dental Department.

The third annual commencement of the Dental Department of the Tennessee Medical College was held in connection with that of the Medical Department, at Staubs' Theatre, Knoxville, Tenn., Thursday evening, March 17, 1892.

After the usual exercises the degree of D.D.S. was conferred on the following persons:

| NAME | RESIDENCE | NAME | RESIDENCE |
|---|---|---|---|
| S. Bennett | Tennessee | E. B. Pennington | Tennessee |
| T. R. Donnelly | Tennessee | G. R. Rouse | South Carolina |
| B. F. Scott | Tennessee | J. G. Foley | Kentucky |
| David Rees | Tennessee | J. H. McCallie | Idaho |

Total, 8.

### Pennsylvania College of Dental Surgery.

The thirty-sixth annual commencement exercises of the Pennsylvania College of Dental Surgery were held at the American Academy of Music, Philadelphia, Pa., on Wednesday evening, March 2, 1892, at 8 o'clock.

The annual address was delivered by Professor Albert P. Brubaker, M.D., D.D.S.

The number of matriculates for the session was two hundred and seven.

The degree of D.D.S. was conferred on the following graduates by L. Minis Hayes, M.D., President of the Board of Corporators:

| NAME | RESIDENCE | NAME | RESIDENCE |
|---|---|---|---|
| C. E. Algeire | New York | Henry Fischer | Germany |
| C. M. Ashton | Pennsylvania | L. H. Franz | Pennsylvania |
| A. R. Atwood | New Jersey | C. E. Foster | New Hamphire |
| Edwin Banton | New York | Emilio Galvis | Colombia, S. A |
| H. Baumgartner | Pennsylvania | Wm. Glading | Pennsylvania |
| Caleb Bird | New York | W. B. Gearhart | Pennsylvania |
| Edith L' Brown | Pennsylvania | W. C. Griffith | Pennsylvania |
| H. S. Brown | Pennsylvania | Geo. F. A. Graf | New York |
| Sylvester Byrne, jr | Pennsylvania | C. H. Green | Delaware |
| Thos. H. Carr | New York | Mayo A. Greenlaw | California |
| D. H. Covert | Canada | W. C. Gutelius | Pennsylvania |
| C. C. Corbiere | California | F. E. Gutelius | Pennsylvania |
| C. J. Chambers | Pennsylvania | A. J. Hamm | Massachusetts |
| S. S. Crow | Missouri | Josiah Hartzell | Ohio |
| John Davenport | Pennsylvania | Mittie Tudor Haley | Virginia |
| A. R. Day | New York | E. B. Heston | Pennsylvania |
| W. H. Deal | New York | Luther Hogarth | Canada |
| George Doerbecker | Illinois | Edwin Hollenback | Pennsylvania |
| Geo. R. Drew | Massachusetts | C. A. Hottenstein | Pennsylvania |
| H. J. Fleming | Pennsylvania | Alice Jarvis | Michigan |

| NAME | RESIDENCE |
|---|---|
| Mary Jaffe | Russia |
| Samuel Johnson | New Jersey |
| M. W. Jennings | Pennsylvania |
| A.H. Keats | Minnesota |
| Mary E. Keyser | Pennsylvania |
| George Kumpf | Canada |
| H. H. Kuhn | Maryland |
| W. H. Lancaster | Connecticut |
| P. L. Longnecker | Pennsylvania |
| M. W. Maratta | Pennsylvania |
| O. J. Marcy | Pennsylvania |
| Jeannie Maguin | Germany |
| W. C. McCarthy | New York |
| G. S. McDowell | Pennsylvania |
| Joe. E. Mitinger | Pennsylvania |
| G. A. Miller | Pennsylvania |
| E. L. Moore | Pennsylvania |
| W. A. May | Canada |
| D. H. Morgan | Ohio |
| T. D. Morrison | Kentucky |
| D. A. Myers | Pennsylvania |
| Girardo Nunez | Colombia, S. A |
| J. C. Nolen | Pennsylvania |
| C. L. Pearson | New York |
| R. B. Pealer | Pennsylvania |
| J. R. Powell | New York |
| Pauline Prime | New York |
| Raul Ramos | Cuba |
| Samuel Rankin | Pennsylvania |
| W. A. Robb | Pennsylvania |
| Joaquin Restrepo | Colombia, S. A |
| J. C. Reynolds | Pennsylvania |
| E. C. Rice | Pennsylvania |
| Oswaldo Ros | Cuba |
| J. H. Ross | Missouri |
| J. W. Ross | Pennsylvania |
| W. J. Roe | Canada |
| J. H. Russell | Pennsylvania |
| W. A. Russell | Pennsylvania |
| J. P. Sager | Pennsylvania |
| Sophie T. Satinover | Roumania |
| F. W. Shepherd | Wisconsin |
| Ivar Siqoveland | Minnesota |
| E. M. Slonaker | Pennsylvania |
| J. H. Slaughter | New Jersey |
| M. W. Snow | Utah |
| O. W. Snow | Utah |
| Martha Sochatzey | Germany |
| Thad. Stine | Pennsylvania |
| M. A. Street | New Jersey |
| C. S. Street | New Jersey |
| E. A. Talmage | Pennsylvania |
| F. W. Tate | New York |
| L. G. Terry | New York |
| John Toprahanian | Turkey |
| J. W. Todd | Pennsylvania |
| Archie V. Toy | Pennsylvania |
| G. A. Vandersluis | Minnesota |
| C. E. Wade | Pennsylvania |
| E. F. Wayne | Pennsylvania |
| J. H. Wardlaw | Canada |
| G. M. Weirich | Pennsylvania |
| E. C. Wiley | Pennsylvania |

Total, 103

### University of Maryland—Dental Department.

The annual commencement exercises of the Department of Dental Surgery of the University of Maryland were held at the Lyceum Theatre, Baltimore, Md., on Thursday, March 17, 1892.

The mandamus was read by the Dean, Prof. Ferdinand J. S. Gorgas, M.D., D.D.S.; the address to the graduates was delivered by Rev. William T. Roberts, of Virginia; and the class oration by H. Janney Nichols, of Virginia.

The number of matriculates for the session was one hundred and twenty-seven.

The degree of D.D.S. was conferred on the following graduates by Hon. S. Teackle Wallis, LL.D., Provost of the University.

| NAME | RESIDENCE |
|---|---|
| W Wolsley Alton | Canada |
| Walter C Anderson | Virginia |
| Fletcher G Asbill | S Carolina |
| Dabney G Barnitz | Virginia |
| Charles F Baylis | New York |
| John C C Beale | Maryland |
| Alexander J Beville | Texas |
| Samuel E Braendle | Canada |
| Winfield S Burd | Pennsylvania |
| Andrew S Burke | Pennsylvania |
| W Bolivar Byers | S Carolina |
| E Marcellus Copenhaver | Virginia |
| W Felton Deekens | Virginia |
| J Harry Deems, jr | Maryland |

| NAME | RESIDENCE | NAME | RESIDENCE |
| --- | --- | --- | --- |
| William W Dennis | Georgia | H Janney Nichols | Virginia |
| John H Diddle | W Virginia | Clyde Sylvanus Payne | California |
| William E Dobson | New York | George C Probst | S Carolina |
| John Lyons Doremus | France | George B Quinlan | New York |
| Eben B Edgers | Vermont | Turner A Ramey | W Virginia |
| Robert W Eicholtz | Pennsylvania | Joseph L Rathie | Virginia |
| Louis Ewig | Switzerland | E Edington Reynolds | New York |
| C Dixie Farriss | Georgia | Jacob Riser | Iowa |
| Lawrence S Fox | N Carolina | Edmund D Shaw | New York |
| Edwin J Gill | N Carolina | James W Simpson | Virginia |
| Eli Harmon Glasscock | Missouri | Will R Simpson | S Carolina |
| George H Hargrove | S Carolina | Harry Blackburn Smith | Bermuda |
| Oscar J Harmon | N Hampshire | Charles B Stouffer | Pennsylvania |
| Lewis E Hess | Maryland | M Emmert Stover | Pennsylvania |
| Frederick C Humberg | Maryland | Arthur O Thomas | S Carolina |
| Hugh Barbour Hutchison | Virginia | William A Thrush | Illinois |
| Benjamin L Jefferson | Georgia | Die P Tipton | Nebraska |
| Silas J Johnson | Virginia | Arminius W Totten | N Carolina |
| B Arthur Jordan | California | William H Van Nostrand | New York |
| James M King | Canada | Harry Van Tassel | South Dakota |
| C Rogers LeFevre | Maryland | Joseph M Veza | Austria |
| J Clinton Macomber | Pennsylvania | Frank Von Wachter | Maryland |
| Thomas Rollins Marshall | Virginia | J Willie Watson | W Virginia |
| Anthony H Mathieu | Maryland | Montgomery Lewis White | Texas |
| W Glenn McGee | S Carolina | Charles G Wiley | Pennsylvania |
| George A McGuire | Canada | Henry A Wilson | Maryland |
| Robert J McHarg | Canada | Edward Kirk Woods | N Hampshire |
| J Morton McIlvain | Maryland | A Watson Woodward | Virginia |
| C Augustus Mitchell | New York | J Harvey Wool | Virginia |
| Harry B Mitchell | New York | | |

Total, 75

### University of California—College of Dentistry.

The annual commencement exercises of the College of Dentistry of the University of California were held at Odd Fellows' Hall, Wednesday evening, December 23, 1891. The term closed December 31st.

The number of matriculates for the session was ninety-eight.

The address on behalf of the Faculty was delivered by Leander Van Orden, jr., M.D.

The degree of D.D.S. was conferred on the following graduates by Prof. Clark L. Goddard, A.M., D.D.S., Chairman of the Faculty:

| NWME | RESIDENCE | NAME | RESIDENCE |
| --- | --- | --- | --- |
| Josephine W. Armstrong | California | Charles Evans Meek | California |
| Charles Franklin Bauer | California | Albert D. E. Milds | California |
| Charles Henry Bell | California | Robert Forrester Millar | California |
| John Millard Blodgett | California | Robert Isaac Moore | California |
| Cecil Corwin | California | Howard Deloss Noble | California |
| D. Carter Elliot | California | Forrest Hoy Orton | Minnesota |
| Philip Foster Frear | California | Frank Harry Phillips | California |
| Charles Lawrence Griswold | California | Harry Griffin Richards | California |
| Charles George Hyde | California | Harold Lawrence ~eager | California |
| Edwin Chandler Hyde | Oregon | Harry Howard Shaw | California |
| William Martin | India | Geo. Newins Van Orden | California |
| John Patrick McCarty | California | Gustavus Adolphus Weyer | California |

Total, 24

### Indiana Dental College.

The thirteenth annual commencement of the Indiana Dental College was held on Tuesday evening, March 1, 1892, at Englishs Opera House, Indianapolis.

A very interesting address on "The Sun", illustrated with fifty sterioptieon views, was delivered by President John, of De Pauw University.

The degree of D.D.S. was conferred on the following graduates:

| NAME. | RESIDENCE | NAME. | RESIDENCE |
|---|---|---|---|
| W. Anderson | Minnesota | D. L. Lucus | California |
| W. G. Burket | Indiana | D. B. Lockhart | Indiana |
| B. F. Batson | Illinois | J. O. Miessen | Indiana |
| G. W. Burch | Nebraska | P. N. Malm | Minnesota |
| C. E. Burket | Indiana | W. J. Morris | Indiana |
| Orlando Burns | Indiana | W. L. McNamara | Ohio |
| J. H. Bloor | Ohio | Chas. B. Pletcher | Indiana |
| W. J. Bradbury | Wisconsin | A. A. Powell | Indiana |
| G. G. Billman | Indiana | E. E. Pierce | Indiana |
| H. M. Brown | Illinois | D. L. Prall | Indiana |
| W. T. Clarke | Texas | P. A. Rood | Indiana |
| Harry Corken | Ohio | Claude V. Runyan | Indiana |
| W. E. Diley | Indiana | M. A. Root | Michigan |
| H. D. Dewar | Michigan | W. B. Ridgeway | Indiana |
| D. A. Elwell | Ohio | Elmer A. Smythe | Indiana |
| G. C. Fleischman | Wisconsin | R. W. Sessions | Indiana |
| W. A. Gant | Indiana | Blaine Sellers | Indiana |
| E. H. Gage | Indiana | J. G. Schneider | Wisconsin |
| J. H. George | Indiana | T. W. Scott | Ohio |
| H. C. Goodrich | Indiana | E. B. Tyler | Indiana |
| C. F. Gray | Indiana | C. W. Throop | Michigan |
| B. F. Gray | Indiana | F. E. Woods | Indiana |
| D. W. Gray | Indiana | Q. H. Woodruff | Indiana |
| W. M. Hall | Indiana | M. L. White | Indiana |
| J. E. Henderson | Indiana | F. Wright | Minnesota |
| W. H. Harp | Illinois | A. T. White | Indiana |
| D. S. Hontz | Indiana | F. Winchester | Michigan |
| W. Z. King | Indiana | Total, 55 | |

### Ohio College of Dental Surgery.

The forty-sixth annual commencement of the Ohio College of Dental Surgery was held at the Odeon, Cincinnati, O., Wednesday, March 9, 1892, at 8 o'clock P. M.

The number of matriculates for the session was one hundred and fifty.

The address was delivered by Dr. W. O. Thompson, President of Miami University, and the Class Oration by Garrett A. Billow, of New Carlisle, Ohio.

The degree of D.D.S. was conferred on the following persons by James Leslie, D.D.S., of the Board of Trustees:

| NAME. | RESIDENCE. |
|---|---|
| John Ray Adair | Kentucky |
| Alexander Scott Ager | Ohio |
| Anthony Lewis Amann | Ohio |
| Charles D. Arthur | Pennsylvania |
| Charles P. Balger | Ohio |
| Isaac Pettit Bell | Canada |
| Porter Adolphus Bereman | Ohio |
| Charles Solomon Beyl | Ohio |
| Garrett Allen Billow | Ohio |
| Will Gavit Bradford | Ohio |
| Louis Arnold Broring | Ohio |
| Harry Lincoln Brown | Illinois |
| Fred. D. Burnham | Ohio |
| Julian Caswell Cavagna | Ohio |
| Henry M. Chaney | Ohio |
| Geo. Amos Chapman | Washington |
| Ch. Campbell Cherryholmes | Nebraska |
| Joseph Boran Cochran | Kentucky |
| Charlie Alvie Cole | Ohio |
| John Lorenzo Conn | Kansas |
| Neclessen S. Cox | Indiana |
| Robert L. Criswell | West Virginia |
| Harrison James Custer | Ohio |
| Miss Hattie A. Dobell | Indiana |
| Elvin Parker Eddy | Ohio |
| William Baker Fahnestock | Ohio |
| Phillip Robert Feigle | Kentucky |
| Orlando Moses Flinn | Indiana |
| Edward Bradley Greenlee | Ohio |
| Alex. Hall | Canada |
| Ernest Rush Hall | Ohio |
| Frank P. Hamilton | Ohio |
| Clement Vernon Hargitt | Ohio |
| Herman Haupt | Germany |
| Homer Thomas Hawkins | Ohio |
| Lonzo Carl Hill | Ohio |
| Horace Anson Holmes | Michigan |
| Curtin Joseph Howe | Pennsylvania |
| Harold Lorenz Jensen | Louisiana |
| Archie Hubert Johnson | Missouri |
| David Saylor Johnson | Pennsylvania |
| Allen John Kimm | Indiana |
| Augustus Fayette Knapp | New York |
| Henry Charles Le Beau | Ohio |
| Robbins Foster Lilly | Ohio |
| George Love | Ohio |
| Will Marquart | Ohio |
| William Harrison McAdow | Ohio |
| Sam. Hamp. McCleery | Pennsylvania |
| Louis Eugene Menuez | Ohio |
| Charles Willett Mills | Ohio |
| George Campbell Minturn | Ohio |
| Leon David Monks | Pennsylvania |
| George Edward Moore | Canada |
| H. Sterling Moore | Ohio |
| Montie A. Morey | Michigan |
| Edward Parker Nugent | Kansas |
| David Cochran Patterson | Kentucky |
| Edwin Auber Peebles | Ohio |
| King Sansom Perry | Pennsylvania |
| Robert Gale Pinney | Missouri |
| Wilber Nathan Priddy | Kansas |
| William Alonzo Pride | Ohio |
| Henry William Radcliff | Wisconsin |
| Frank Benjamin Rees | Ohio |
| Oliver Taylor Robertson | Ohio |
| James Holton Robinson | Canada |
| Edwin Launder Ross | Ohio |
| Daniel Ulrich Ruegsegger | Ohio |
| Rudolph Schmid | Switzerland |
| Albert Edward Sexton | Indiana |
| Charles William Sodders | Ohio |
| Talmon H. Speece | Ohio |
| Henry Marion Smith | Minnesota |
| Charles Willard St. Clair | Ohio |
| James Berry Stewart | Ohio |
| William Harold Tenney | Ohio |
| Clyde Everett Townley | Pennsylvania |
| Joseph Armstred Turner | Ohio |
| Rees L. H. Turner | Missouri |
| Francis Marion Van Buskirk | Ohio |
| Horatio Frank Vandervort | Ohio |
| Thomas Corwin White | Ohio |
| John C. Wilde, Jr | Michigan |
| William Elmore Wilkinson | Ohio |
| Ellsworth Williams | Indiana |
| Franz Ellias Willison | Michigan |
| Mrs. Mellie C. Winslow | Indiana |
| Sherman Tecumseh Yaple | Ohio |

Total, 89

### New York College of Dental Surgery.

The twenty-sixth annual commencement of the New York College of Dentistry was held in Chickering Hall, New York City, on Thursday evening, March 10, 1892.

The number of matriculates for the session was two hundred and seventy-three.

The Valedictory was delivered by Henry P. King, D.D.S., of the graduating class, and the Address to the graduates by Wm. H. McElroy, Esq.

The degree of D.D.S. was conferred on the following persons by Wm. T. La Roche, D.D.S., Vice-President of the Board of Trustees:

NAME.
John Patrick Burke.
Miska Lipot Braun.
Edward Santley Butler.
Walter Benney.
Frederick Brueckner.
George Francis Barrett.
Eugene Bonilla Y Cuibas.
James Edward Byrne.
Henry Emile Bischof.
John Francis Buckley.
Carl Rudolph Otto Bickel.
Albert William Crosby.
John Phillip Cromwell.
Edward Archibald Crostic.
George Edward Christie.
Martin Lawrence Collins.
Nelson Millard Chitterling.
Louis Bristol Daboll.
Harry Clay Derby.
Richard Francis Doran.
Anthony Chas. Durschang.
Joseph Fuld.
H. Clay Richardson Ferris.
Finn Fosheim.
Edgar Ozias Goodell.
John Francis Goger.
Walter Harris Gardner.
William Henry Garrett.
Dexter Glennon Gordon.
Charles Frank Guntner.
Charles Casselman Gibson.
Joseph Gluck.
Joseph Harvitt.
John Henry Hughes.
Wm. Henry Moore Hamlett.
Peter James Heffern.
Otto George Hoffman.
Orion Perseus Howe.
Henry Dryer Hatch.
Byron Edward Joubert.
George Washington Koles.
Eli Koles.
Harry Taylor Kelsay.
Isaac Kroch.

NAME.
Henry Palmer King.
Henry Albert Kregeloh.
Ernest August Kolling.
Frank Belknap Long.
Frank Leroy Lockwood.
Alfred Tennyson Lockwood.
W. Hawthorne McCutcheon.
Miguil Romon Mangual.
Frank Lester Munsell.
Augustus MacCollom, Jr.
Julius Adolph Mayer.
Alonzo Silas Mead.
Edward William McNeil.
Frederick Thos. Murlless, Jr.
Henry Alfred Neech.
Frederick Smith Parsons.
Henry Amon Parmentier.
George Elbert Reynolds.
George Alphonse Roussel.
Samuel Schnaper.
Frank Schroeder.
Aug. Vancortlandt Stebbins.
Henry Josiah Stacpoole.
Jacob Schn[illegible]ier.
E. Warren Sylla.
Henry Gustav Schroeder.
Edward John Moritz Seebold.
Engelbert Stoetzer.
Mario Tolosa Y Polidura.
Ezra Oakley Taylor.
Zebulon Scriven Taylor.
George Vande Verg.
Orwill Van Wickle.
George Putnam Willis.
Edwin Chapin Wallace.
Henry Lamont Wheeler.
Frank Jackson Woodworth.
Augustine Joseph Walsh.
Edgar Williams.
Willie Jackson Ward.
Leon Jabez Weeks.
Harry Fones Whitter.
Floyd Marcus Zelie.
Total, 87

### Vanderbilt University—Dental Department.

The thirteenth annual commencement exercises of the Dental Department of the Vanderbilt University were held at the Vendome Theatre, Nashville, Tenn., February 24, 1892.

The number of matriculates for the session was one hundred and twenty-six.

The Class Oration was delivered by C. J. Washington, D.D.S., of Tenn., and the Faculty address by Dr. C. S. Stockton, of New Jersey.

The degree of D.D.S. was conferred by L. C. Garland, the Chancellor, on the following persons :

| NAME. | RESIDENCE | NAME. | RESIDENCE |
|---|---|---|---|
| V. W. Alexander | New York | C. C. Evans | Illinois |
| C. R. Adams | Mississippi | C. Eshleman | Iowa |
| C. S. Allred | Alabama | T. A. Fayette | Alabama |
| J. M. Ashburn | Tennessee | F. B. Gaither | North Carolina |
| G. M. Brown | Michigan | C. B. Graham | South Carolina |
| L. Bland | Louisiana | W. I. Hale | Alabama |
| A. E. Brown | Texas | W. L. Hansbro | Tennessee |
| F. Bartell | Illinois | A. C. Jones | Tennessee |
| J. A. Beavers | Alabama | R. A. Jones, Jr | Alabama |
| J. R. Beach | Tennessee | W. I. Johnson | Alabama |
| J. S. Brown | Mississippi | E. L. Kendrick | Alabama |
| F. K. Barefield | Mississippi | B. E. Kidd. | Alabama |
| J. P. Corley | Alabama | O. G. Mingledorff | South Carolina |
| R. Z. Chapman | Alabama | T. W. McKell | Mississippi |
| R. H. Carratle | Iowa | M. B. McCrary | Tennessee |
| J. J. Cook | Michigan | J. M. Murphree | Alabama |
| D. P. Cook | Kentucky | C. W. Mathison | Alabama |
| S. C. Cawthorn | Florida | J. M. Millen | Tennessee |
| W. J. Dillard | Texas | G. Minnick. | Illinois |
| J. S. Dalton | Missouri | A. J. Newcomer | Illinois |
| S. K. Davidson | Kentucky | J. B. Penny | Missouri |
| E. H. Dennison | Connecticut | J. H. Palm | Germany |
| W. H. Powell | Louisana | F. O. H. Thiele | Germany |
| A. L. Pedigo | Texas | C. J. Washington | Tennessee |
| M. D. Steele | Louisiana | V. B. Warrenfells | Virginia |
| C. A. Sevier | Tennessee | J. D. Wise | Alabama |
| R. Sanderson | Alabama | F. P. Ward | Alabama |
| F. W. Simons | Texas | W. L. Weatherby | Mississippi |
| W. K. Slater | Tennessee | N. F. Weatherby | Mississippi |
| C. C. Sims | Arkansas | H. W. Walker | Georgia |
| N. W. Sherman | Tennessee | V. H. Ward | Mississippi |
| M. O. Sallee | Kentucky | V. A. Williams | California |
| H. E. Spencer | Mississippi | H. Wiggins | Texas |
| W. S. Taylor | Kentucky | A. Walker | Georgia |
| R. E. Thornton | Georgia | C. M. Walton | Tennessee |

Total, 70

### American College of Dental Surgery.

The sixth annual commencement exercises of the American College of Dental Surgery were held at Hooley's Theatre, Chicago, Ills., on Wednesday, March 9, 1892, at 2 P. M.

The valedictory address was delivered by H. E. Myers, D.D.S.

The degree of D.D.S. was conferred on the following members of the graduating class:

| NAME | NAME |
|---|---|
| W C Brown | E A Fritis |
| F F Brown | R M Grimes |
| A J Bacon | A L Gilmer |
| W T Corwith | V C Garratt |
| C M Cody | W S Graves |
| C I Chase | W S Harter |
| George Collins | R V Hurdle |
| C L Crossman | E C Hoffman |
| A E Crum | O C Hall |
| I B Carolus | Caroline L Hartt |
| L M Darling | J Hetu |
| P E Douglass | C S Irwin |
| C L Davis | Jennie Loretto Kelly |
| H W Davenport | H P Loomis |

| NAME | NAME |
|---|---|
| C L H Lennmalm | Adelaide F Rix |
| W H Lillibridge | W T Rogers |
| J A Messenger | W E Sturmberg |
| H E Myers | B R Simons |
| Geo W Mills | A E St John |
| W N McCay | W R Smith |
| M G E Marshall | A O Stutenroth |
| C S Marshall | H F. Stempel |
| W T Morris | C F Smith |
| A S Marshall | Lucy M Scott |
| W A Nelson | George Steele |
| J M Oaks | Florence E Thompson |
| I J Pierce | C C Trowbridge |
| Edgar Palmer | L A Tidball |
| Josephine D Pfeifer | H H Von Lackum |
| W E Pilcher | S A Wilson |
| S T Rice | I C Ward |
| Fanny M Rowley | H J Wallin |
| F C Ross | V S Wisner |

Total, 66

### Homœopathic Hospital College—Dental Department

The first annual commencement of the Dental Department of the Homœopathic Hospital College, of Cleveland, O., was held in connection with that of the other departments in the College Building on March 22, 1892.

The number of matriculates for the session was fifteen.

The degree of D.D.S. was conferred on the following graduates:

| NAME | NAME |
|---|---|
| P. W. Murton | J. M. Clyne, M.D |
| C. L. Kelsey | G. E. Bishop |
| C. S. Geer, M.D | W. E. Root |

Total, 6.

Doctor of Medicine—Honorary—was conferred upon S. B. Dewey, J. E. Robinson, H. Barnes and L. P. Bethel.

THE number of men working on the Exposition buildings is now more than six thousand. On some of the buildings work is proceeding day and night.

PRINCE GEORGE of Wales, who, if he lives, will some day be king of England, will visit the Exposition, a cablegram from London announces.

### Northwestern University—Dental Department.

The commencement exercises of the Dental Department of the Northwestern University were held in connection with those of the Medical Department in Central Music Hall, Chicago, Ill., on Tuesday, April 26, 1892.

The number of matriculates for the session was fifty-six.

The faculty address was delivered by Prof. J. H. Hollister, A.M., M.D.

The degree of D.D.S. was conferred on the following graduates by Henry Wade Rodgers, LL.D., president of the University:

| NAME | NAME |
|---|---|
| Charles Martin Baldwin | Alvah Bradmon Graham |
| James Lewis Blish | William Fielding Garnett |
| William Leonard Barnes | George Byron Hiller |
| Joseph Free Baird | William Edward Merritt |
| Edwin Morgan Chapman | Augustus Gorman Miller |
| Lewis Samuel Celley | Samuel Thomas Mitchell |
| Adam William Feltmann | Clifford Murry Roberts |
| John Lloyd Foster | George Everett Warren |
| William Alfred Grove | Doctor Merritt Wilcox |

Total, 18.

A FEW years ago the Dental Section was formed in the American Medical Association, and because many practicing dentists were graduates of dental colleges and not of recognized medical schools, it was necessary that they should have their own autonomy. This has worked very satisfactorily and the Dental Section has been a great power for good in encouraging dentists to complete their professional education by taking the regular degree of doctor of medicine as well as that of dentistry, so that they may come into closer harmony with the medical profession as recognized specialty practitioners.

DR. H. P. C. WILSON, of Baltimore, says that of the thousand patients who have come to him suffering from the opium habit nearly all have been led into it by the attending physician. He thinks no diseased condition, except advanced and rapidly fatal cancer, justifies the habitual use of opium for the relief of pain.—*American Lancet.*

